# Data of nearby space objects using SIMBAD astronomical database

**DOI:** 10.1016/j.dib.2023.108943

**Published:** 2023-02-01

**Authors:** Bahram Kalhor

**Affiliations:** Shahid Beheshti University, Faculty of Electrical and Computer Engineering, Iran

**Keywords:** Astronomical database, Redshift, Temperature of space objects, Parallax distance

## Abstract

Although SIMBAD Astronomical Database lets us write our query to extract data, there are some problems. Max record number in each query is too low. Also, repeating the name of stars in different records is a big problem. Hence, we wrote a script and executed it at different distances. Also, we wrote a program for grouping data and deleting repeated records. The article represents the distance, temperature, and Redshift of 93,060 nearby space objects, including stars, quasars, white dwarfs, and carbon stars. The objects' temperatures are between 671 and 99,575 K, and the distances of the objects are between 413.13 and 0.5 (mas). We have retrieved this information from almost 2,200,000 records. In addition, we have added two new columns for providing equivalent distances in the light year and peak frequency of the black body. All data are in a simple table in a Microsoft Access Database and a copy in the Excel. We have excluded data from space objects whose temperature doesn't exist and space objects whose Redshift is less than zero (Blueshift). The SIMBAD Astronomical Database provides the distance of the space objects using the parallax method. The advantage of choosing nearby stars is using the Parallax method for calculating the distance of the stars, which is more precise than other methods. The Parallax data help us to investigate space objects in a no-expansion universe. We can use this data in many different investigations. Finding a correlation between temperature and Redshift of stars, investigating the nature of nearby space objects with Redshift higher than 1, and investigating the origin of the Quantum Redshift in a no expansion universe using parallax distance are some useful usages of this data.

**Specifications Table**

**Table:** 

Subject	Astronomy
Specific subject area	As a natural science, astronomy studies celestial objects and phenomena, using mathematics, physics, and chemistry to explain their existence.
Type of data	Table, Figure
How the data were acquired	We have used SIMBAD's TAP (Table Access Protocol) service (http://simbad.u-strasbg.fr/simbad/sim-tap) to extract the data of the nearby space objects. SIMBAD is the acronym for: Set of Identifications, Measurements and Bibliography for Astronomical Data and TAP is a protocol which describes a way to query data tables. The SIMBAD's TAP service lets us write our script and execute it for extracting necessary data. We wrote a script with some conditions; the source of the script is represented in a text file. We have excluded data records with a temperature equal to or less than 100 K. Also, the article has excluded the space objects with blueshift, and we have used the average temperatures of the space objects. Because of the limitation of the number of output records in each execution, we executed the script many times in different distance and temperature ranges. We merged 1.4 million records of the space objects at different distances with 930,000 records of the hottest nearby space objects from the SIMBAD Astronomical Database. Then we wrote a program for deleting repeated records and merging them into a single table in the Microsoft Access database. At next stage, the unit of distance is converted from parallax to the light-year. Finally, we extracted information of 93,060 space objects, including stars, quasars, white dwarfs, and carbon stars. Final data is saved in a Microsoft Access table. Also, the source of the script is in a text file. Queries are by default written in ADQL (Astronomical Data Query Language). It is possible for researchers to retrieve data by writing a script into “ADQL query to execute” section in the http://simbad.u-strasbg.fr/simbad/sim-tap.
Data format	Table, Graph, Figure
Description of data collection	We extracted this data for testing the Quantum Redshift theory. Hence, we excluded data of space objects with the Redshift lower than zero. Also, we did not add space objects whose temperature did not exist or who were very cold.
Data source location	http://simbad.u-strasbg.fr/simbad/sim-tap
Data accessibility	All data are in a simple table in a Microsoft Access Database. Also, a copy of the data is represented in a excel file. A text file includes basic script for downloading data. Repository name: figshare.com Data identification number: 20099951 Direct URL to data: https://figshare.com/articles/dataset/Nearby_Space_Objects_Name_Distance_Redshift_Temperature/20099951
Related research article	Impact of the temperature of stars on their Redshift.

## Value of the Data

• Researchers can use this data to study new theories about the origin of the Redshift in a no-expansion universe. Also, they can understand the nature of nearby space objects with high Redshift and represent dark matter candidates.
• Anyone who will work on reliable data for testing theories about dark energy, dark matter, Quantum Redshift, Quantum Cosmic Microwave Background [1], and other astronomical subjects can use this data.
• This data could be used for statistical studies [2] and finding a correlation between distance, temperature, and Redshift.
• This data could be used for testing the predictions of the Quantum Structure of Electromagnetic Waves in the real world. In this theory, Redshift occurs because of sharing of the energy between light periods.
• This data could be used by the researchers who work on combining the basics of quantum physics, special relativity, and new concept for the quantum structure of the electromagnetic waves.

## 1 Data Description

The data article presents a database, including a table of 93,060 nearby space objects. The source of this data is the SIMBAD Astronomical Database which is the reference database for the identification and bibliography of astronomical objects. It is a database consisting of object identifications, information about the objects' basic properties, a bibliography, and observations of selected objects. It is developed and maintained by CDS (The Centre de Données Astronomiques de Strasbourg). Several institutes contribute to the database's contents [3].

The table consists of 6 data fields: object name, distance (mas), temperature, and Redshift have been retrieved directly from the SIMBAD Astronomical Database. In addition, the peak frequency of the black body and distance in the Light Year have been calculated using temperature and parallax distance, respectively. The advantages of the parallax distance are precision and reliability. These distances could be used to test the formulas and predictions of the theories that disagree with the expansion of the universe. The field ID isn't a data field. It has been added as the primary key to use in future designs and queries.

For testing the correctness of the data and getting more information about the space object we can use the “Object Name” field of the table. The format of the source page of the object in the SIMBAD Astronomical Database is: http://simbad.u-strasbg.fr/simbad/sim-id?Ident=ObjectName.

If we replace the “ObjectName” at the end of the above link with the real object name in the table, we will redirect to the appropriate page on the SIMBAD website. For instance, using NGC 4688 at the end of the link instead of the “ObjectName” redirect us to the information page of the NGC 4688. The temporary link format is given by: http://simbad.u-strasbg.fr/simbad/sim-id?Ident=NGC 4688.

We should replace “%20” instead of the blank in the name of the object to get a complete URL. The final URL is given by: http://simbad.u-strasbg.fr/simbad/sim-id?Ident=NGC%204688.

**Fig. 1:**
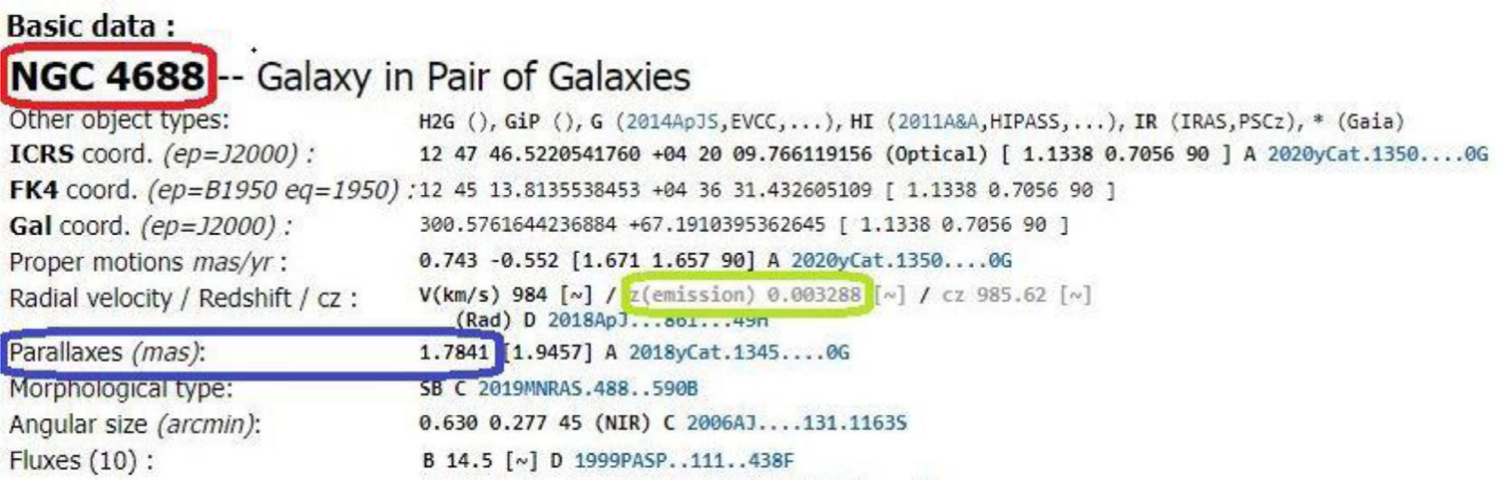
SIMBAD Astronomical Database. Information page of the NGC 4688. In the red box is the Object Name, in the blue box is the Parallax Distance, and in the green box is the Redshift.

Fig. 1 demonstrates the location of each data on the SIMBAD website. The Object Name is in the red box, the parallax distance is in the blue area, and the Redshift of the object is in the green box.

There are some nearby space objects with very high Redshift. There are 41 stars with z > 1, and almost 200 stars have z > 0.001. Table 1 shows nearby space objects with the highest Redshift. Column 1 is the name of the space object, columns 2 and 3 are the distance from the earth, column 4 is the Redshift of the stars, column 5 is the temperature, and column 6 is the peak frequency of the spectrum of the space object according to the Planck's diagram [4], [5], [6], [7].

**Table 1:** Highest Redshift: Top 40 nearby space objects with highest Redshift (z>0 and temperature>100).

Object Name	Distance (mas)	Distance (L.Y)	Z	Temperature (K)	Peak Frequency (Hz)
LSPM J1247+0646	1.91E+01	1.71E+02	3.64E+00	5.65E+03	3.32E+15
SDSS J154213.47+034800.4	6.81E+00	4.78E+02	2.73E+00	1.57E+04	9.21E+15
SDSS J172045.37+561214.9	2.03E+00	1.60E+03	2.72E+00	4.84E+04	2.84E+15
EGGR 561	1.83E+01	1.78E+02	2.72E+00	1.48E+04	8.69E+15
KUV 03292+0035	9.03E+00	3.61E+02	2.72E+00	2.67E+04	1.57E+15
SDSS J090746.83+353821.4	3.27E+00	9.97E+02	2.70E+00	1.21E+04	7.12E+15
SDSS J001939.93+221824.7	1.18E+00	2.76E+03	2.39E+00	8.27E+03	4.86E+15
SDSS J083226.57+370955.4	8.46E+00	3.85E+02	2.27E+00	1.01E+04	5.93E+15
SDSS J080623.77+430519.9	5.61E+00	5.81E+02	2.26E+00	1.06E+04	6.25E+15
SDSS J214631.15-010644.9	4.06E+00	8.03E+02	2.26E+00	1.08E+04	6.38E+15
SDSS J083011.35+383940.4	9.30E+00	3.50E+02	2.26E+00	1.01E+04	5.95E+15
SDSS J145602.80-010952.6	9.42E+00	3.46E+02	2.25E+00	1.01E+04	5.93E+15
SDSS J213507.72-071655.6	7.91E+00	4.12E+02	2.25E+00	6.71E+03	3.95E+15
SDSS J091602.55+002234.8	3.62E+00	9.00E+02	2.25E+00	1.04E+04	6.10E+15
SDSS J090746.33-004640.8	4.26E+00	7.66E+02	2.25E+00	1.13E+04	6.64E+15
SDSS J095949.08+535646.3	3.13E+00	1.04E+03	2.24E+00	1.03E+04	6.06E+15
PHL 1266	6.07E+00	5.37E+02	2.24E+00	1.04E+04	6.09E+15
SDSS J010442.19-084343.9	8.00E+00	4.07E+02	2.24E+00	1.01E+04	5.94E+15
SDSS J135205.59+514930.5	8.90E+00	3.66E+02	2.24E+00	1.01E+04	5.93E+15
SDSS J091417.92+080841.5	5.96E+00	5.47E+02	2.23E+00	7.33E+03	4.31E+15
SDSS J141715.39+495639.9	2.77E+00	1.18E+03	2.23E+00	1.09E+04	6.38E+15
SDSS J114648.63+570945.8	3.00E+00	1.09E+03	2.23E+00	8.09E+03	4.76E+15
SDSS J111432.16+630914.4	2.55E+00	1.28E+03	2.23E+00	1.04E+04	6.12E+15
SDSS J144408.76+024327.8	3.65E+00	8.94E+02	2.22E+00	7.30E+03	4.29E+15
SDSS J102517.27+572047.4	3.80E+00	8.57E+02	2.22E+00	1.04E+04	6.13E+15
SDSS J112353.92+594725.8	6.49E+00	5.02E+02	2.22E+00	1.13E+04	6.64E+15
SDSS J173106.82+533112.0	3.48E+00	9.36E+02	2.22E+00	1.08E+04	6.35E+15
SDSS J080332.75+433955.1	6.08E+00	5.36E+02	2.21E+00	1.02E+04	5.99E+15
SDSS J115944.37+563606.9	3.56E+00	9.16E+02	2.21E+00	1.05E+04	6.16E+15
SDSS J231629.37-093845.6	1.01E+01	3.22E+02	2.20E+00	1.01E+04	5.93E+15
SDSS J222629.42+004254.1	6.63E+00	4.92E+02	2.20E+00	1.02E+04	6.00E+15
SDSS J090051.91+033149.3	8.60E+00	3.79E+02	2.19E+00	1.01E+04	5.94E+15
LP 708-404	1.40E+01	2.33E+02	2.18E+00	1.01E+04	5.93E+15
SDSS J100441.23+620746.9	3.25E+00	1.00E+03	2.18E+00	1.02E+04	6.00E+15
PB 6723	8.31E+00	3.92E+02	1.80E+00	1.02E+04	5.98E+15
SDSS J125205.66+662902.8	4.24E+00	7.69E+02	1.78E+00	1.26E+04	7.42E+15
SDSS J004358.93+143305.7	3.99E+00	8.18E+02	1.78E+00	1.05E+04	6.20E+15
SDSS J095137.60+624348.7	5.92E+00	5.51E+02	1.77E+00	1.02E+04	5.98E+15
SDSS J171356.04+611332.7	5.40E+00	6.04E+02	1.76E+00	1.17E+04	6.86E+15
SDSS J100817.03+434931.7	6.84E+00	4.76E+02	1.76E+00	1.19E+04	7.02E+15
SDSS J124536.66+042823.8	1.91E+01	1.71E+02	3.64E+00	5.65E+03	3.32E+15

Table 2 shows nearby space objects with the highest temperature and their highest peak frequency according to the Planck's diagram. The equation is given by:

$$f_{peak}=\;5.881468277945619E10\;*\;T$$

**Table 2:** Highest temperature: Top 40 nearby space objects with highest temperature (z>0 and temperature>100).

Object Name	Distance (mas)	Distance (L.Y)	Z	Temperature (K)	Peak Frequency (Hz)
SDSS J145545.58+041508.6	1.66E+00	1.97E+03	1.96E-04	9.96E+04	5.85E+15
SDSS J092651.43+254859.0	1.50E+00	2.17E+03	8.19E-04	9.96E+04	5.85E+15
2QZ J133710.1-002644	3.15E+00	1.03E+03	3.00E-04	9.57E+04	5.63E+15
SDSS J132858.19+590851.0	6.66E+00	4.89E+02	7.86E-01	9.45E+04	5.56E+15
SDSS J200646.50-124410.9	1.11E+00	2.94E+03	9.34E-05	9.34E+04	5.49E+15
NGC 4688	1.78E+00	1.83E+03	3.23E-03	8.95E+04	5.26E+15
SDSS J211607.27+004503.2	3.91E+00	8.34E+02	5.90E-04	8.89E+04	5.22E+15
SDSS J222203.33-003138.1	1.48E+00	2.21E+03	2.84E-05	8.84E+04	5.20E+15
SDSS J100612.78+252833.6	2.46E+00	1.32E+03	1.20E-04	8.73E+04	5.13E+15
SH 2-216	7.94E+00	4.11E+02	4.79E-05	8.70E+04	5.11E+15
SDSS J161613.09+252012.6	1.51E+00	2.16E+03	9.51E-04	8.66E+04	5.09E+15
KUV 03459+0037	2.52E+00	1.29E+03	2.70E-04	8.64E+04	5.08E+15
SDSS J020158.87+132349.5	6.19E-01	5.26E+03	1.08E-03	8.39E+04	4.93E+15
SDSS J160236.07+381950.5	1.11E+00	2.93E+03	3.00E-04	8.29E+04	4.87E+15
PN A66 7	2.02E+00	1.61E+03	6.00E-05	8.27E+04	4.86E+15
SDSS J082153.01+190659.1	1.17E+00	2.78E+03	8.78E-04	8.22E+04	4.83E+15
HZ 34	1.65E+00	1.98E+03	8.67E-05	8.22E+04	4.83E+15
Ton 309	3.00E+00	1.09E+03	1.60E-04	8.22E+04	4.83E+15
PB 7489	1.91E+00	1.71E+03	1.50E-04	7.88E+04	4.63E+15
SDSS J101700.39+190110.1	1.18E+00	2.77E+03	3.63E-05	7.82E+04	4.60E+15
SDSS J105555.23+484739.8	1.22E+00	2.67E+03	1.70E-04	7.74E+04	4.55E+15
PG 1543+454	2.06E+00	1.58E+03	1.00E-05	7.73E+04	4.54E+15
WD 2121-076	2.37E+00	1.38E+03	2.40E-04	7.66E+04	4.50E+15
SDSS J074632.00+415210.2	1.35E+00	2.42E+03	7.90E-04	7.66E+04	4.50E+15
CD-45 5058	1.71E+00	1.91E+03	1.67E-05	7.59E+04	4.46E+15
SDSS J101619.80-020258.2	2.80E+00	1.16E+03	6.00E-05	7.55E+04	4.44E+15
LB 651	1.80E+00	1.81E+03	8.54E-06	7.51E+04	4.41E+15
SDSS J083838.52+065240.1	1.47E+00	2.22E+03	7.71E-05	7.46E+04	4.38E+15
TK 1	1.87E+00	1.74E+03	1.40E-04	7.43E+04	4.37E+15
SDSS J173824.64+581801.8	1.17E+00	2.79E+03	1.10E-04	7.41E+04	4.36E+15
SDSS J095229.03+150350.5	1.09E+00	3.00E+03	4.75E-05	7.38E+04	4.34E+15
SBSS 1317+601	1.06E+00	3.08E+03	6.41E-05	7.28E+04	4.28E+15
SDSS J074520.05+234223.8	1.08E+00	3.02E+03	5.50E-04	7.27E+04	4.27E+15
SDSS J162514.88+302610.8	1.58E+00	2.07E+03	4.00E-04	7.27E+04	4.27E+15
EC 13123-2523	2.39E+00	1.36E+03	5.37E-05	7.26E+04	4.27E+15
HS 0951+3620	1.11E+00	2.93E+03	2.30E-04	7.26E+04	4.27E+15
SDSS J234737.43+135131.0	6.49E+00	5.02E+02	1.06E+00	7.20E+04	4.23E+15
SDSS J085148.55+095035.2	1.40E+00	2.33E+03	7.40E-04	7.13E+04	4.19E+15
SDSS J153127.86+133917.4	1.27E+00	2.57E+03	1.57E-04	7.12E+04	4.19E+15
HS 2246+0640	1.47E+00	2.22E+03	2.50E-05	7.09E+04	4.17E+15

**Fig. 2:**
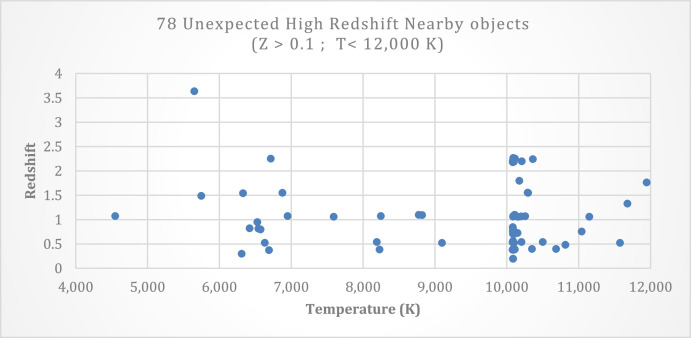
Distribution of 78 high Redshift nearby objects with temperatures between 4000 and 14000 K.

The table in the database can be divided into two separate categories based on the temperature of the objects in space. In the first category, the objects have temperatures close to 6,600 K. The second category contains objects with temperatures near 10,000 K. Fig. 2 shows the distribution of the highest Redshift for objects with temperatures between 4,000 and 12,000 K. It is evidence that there is a high chance of finding unexpected high Redshift objects around 10,000 K.

The distance of these stars can be achieved by searching it in https://gea.esac.esa.int/archive/, based on its name.

Table 3 shows the possibility of finding unexpected high Redshift stars in the special range of the temperatures [8]. In the Table 3, column 1 is the range of temperature, column 2 is the number of the space objects whose temperature belongs to the range of the temperature range, and column 3 is the number of objects whose Redshift is higher than 0.001.

**Table 3:** Number of high Redshift space objects in the different temperature.

Temperature (K)	Number of space objects	Number of high Redshift space objects (z>0.001)
Less than 4000	5,555	26
4000-6000	40,438	44
6000-8000	11,248	32
8000-10000	945	8
10000-12000	387	66
Greater than 12000	1,161	23

Table 4 shows nearby space objects with the lowest distance from the earth out of the solar system.

Although Proxima Centauri is the nearest star to the earth, it is not in the table because of z<0.

Name: Proxima Centauri Distance (mas): 768.0665 z: -7.471556611649E-5

This observational data supports the Quantum Redshift (QR) theory. Data of 93,060 nearby space objects [8] shows a gradual increase in the amount of the lost quanta energies of the space objects with larger distance. The QR theory not only can describe the reason for the Redshift, but also prove the higher rate of increasing Redshift of the distant objects. Hence, the Quantum Redshift rejects accelerating expansion of the universe and dark energy.

On the other hand, the Quantum Redshift is the reason for the Cosmic Microwave Background (CMB). In the QCMB (Quantum Cosmic Microwave Background) electromagnetic waves of all objects will be converted to the CMB over time [1].

**Table 4:** Lowest distance: Top 40 nearby space objects with lowest distance from the earth (z>0 and temperature>100).

Object Name	Distance (mas)	Distance (L.Y)	Z	Temperature (K)	Peak Frequency (Hz)
Wolf 359	4.13E+02	7.89E+00	6.44E-05	2.94E+03	1.73E+15
G 272-61B	3.72E+02	8.76E+00	9.67E-05	3.17E+03	1.86E+15
* eps Eri	3.11E+02	1.05E+01	5.46E-05	5.09E+03	2.99E+15
HD 217987	3.04E+02	1.07E+01	2.94E-05	3.69E+03	2.17E+15
HD 173740	2.84E+02	1.15E+01	3.68E-06	3.35E+03	1.97E+15
V* GQ And	2.81E+02	1.16E+01	3.67E-05	3.35E+03	1.97E+15
V* GX And	2.81E+02	1.16E+01	3.94E-05	3.60E+03	2.12E+15
G 51-15	2.79E+02	1.17E+01	1.80E-04	2.94E+03	1.73E+15
V* YZ Cet	2.69E+02	1.21E+01	9.37E-05	3.12E+03	1.83E+15
BD+05 1668	2.63E+02	1.24E+01	6.08E-05	3.35E+03	1.97E+15
HD 33793	2.54E+02	1.28E+01	8.18E-04	3.62E+03	2.13E+15
V* AX Mic	2.52E+02	1.29E+01	6.86E-05	3.69E+03	2.17E+15
Ross 614	2.43E+02	1.34E+01	5.57E-05	3.10E+03	1.82E+15
Wolf 28	2.32E+02	1.41E+01	8.78E-04	6.15E+03	3.62E+15
HD 225213	2.30E+02	1.42E+01	8.52E-05	3.49E+03	2.05E+15
Wolf 424	2.28E+02	1.43E+01	3.34E-06	3.07E+03	1.80E+15
G 208-45	2.15E+02	1.52E+01	1.81E-05	3.06E+03	1.80E+15
G 208-44	2.13E+02	1.53E+01	1.00E-05	2.92E+03	1.72E+15
BD+44 2051	2.06E+02	1.58E+01	2.29E-04	3.65E+03	2.15E+15
BD+44 2051B	2.04E+02	1.60E+01	2.34E-04	3.02E+03	1.78E+15
HD 204961	2.01E+02	1.62E+01	4.39E-05	3.47E+03	2.04E+15
BD+20 2465	2.01E+02	1.62E+01	4.15E-05	3.48E+03	2.05E+15
V* EV Lac	1.98E+02	1.65E+01	9.57E-07	3.29E+03	1.93E+15
G 99-49	1.92E+02	1.70E+01	1.01E-04	3.21E+03	1.88E+15
G 9-38	1.90E+02	1.72E+01	4.34E-05	3.12E+03	1.83E+15
LP 656-38	1.86E+02	1.75E+01	1.41E-04	3.20E+03	1.88E+15
HD 119850	1.84E+02	1.77E+01	5.27E-05	3.65E+03	2.15E+15
EGGR 180	1.81E+02	1.80E+01	6.67E-06	7.13E+03	4.19E+15
G 175-34	1.80E+02	1.81E+01	9.61E-05	3.25E+03	1.91E+15
LP 816-60	1.78E+02	1.83E+01	5.44E-05	3.27E+03	1.92E+15
2MASSI J1835379+325954	1.76E+02	1.85E+01	2.80E-05	2.49E+03	1.46E+15
HD 36395	1.75E+02	1.86E+01	2.88E-05	3.77E+03	2.21E+15
HD 42581	1.74E+02	1.88E+01	1.58E-05	3.77E+03	2.22E+15
Ross 47	1.73E+02	1.89E+01	3.53E-04	3.23E+03	1.90E+15
HD 131977	1.70E+02	1.92E+01	9.00E-05	4.63E+03	2.72E+15
HD 180617	1.69E+02	1.93E+01	1.20E-04	3.58E+03	2.10E+15
VB 10	1.69E+02	1.93E+01	9.67E-05	2.70E+03	1.59E+15
HD 131976	1.69E+02	1.93E+01	8.64E-05	3.84E+03	2.26E+15
* eta Cas	1.68E+02	1.94E+01	2.80E-05	5.89E+03	3.46E+15
* 36 Oph A	1.68E+02	1.94E+01	1.47E-06	5.13E+03	3.02E+15

## 2 Experimental Design, Materials and Methods

We have used SIMBAD TAP Service for extracting data. The TAP Service provides an environment for writing complex queries and extracting data in different formats. The direct link to the TAP Service is: https://simbad.cds.unistra.fr/simbad/sim-tap.

**Fig. 3:**
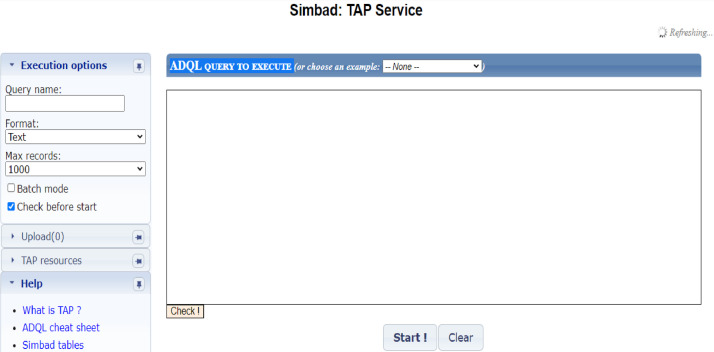
TAP Service environment.

Fig. 3 demonstrates the page of the TAP Service. On the left side of the page, we can specify the format of the output data and the number of the output records. At the center of the page, just below the “ADQL query to execute” there is an empty box where we can write our scripts and run them by clicking the start button.

**Fig. 4:**
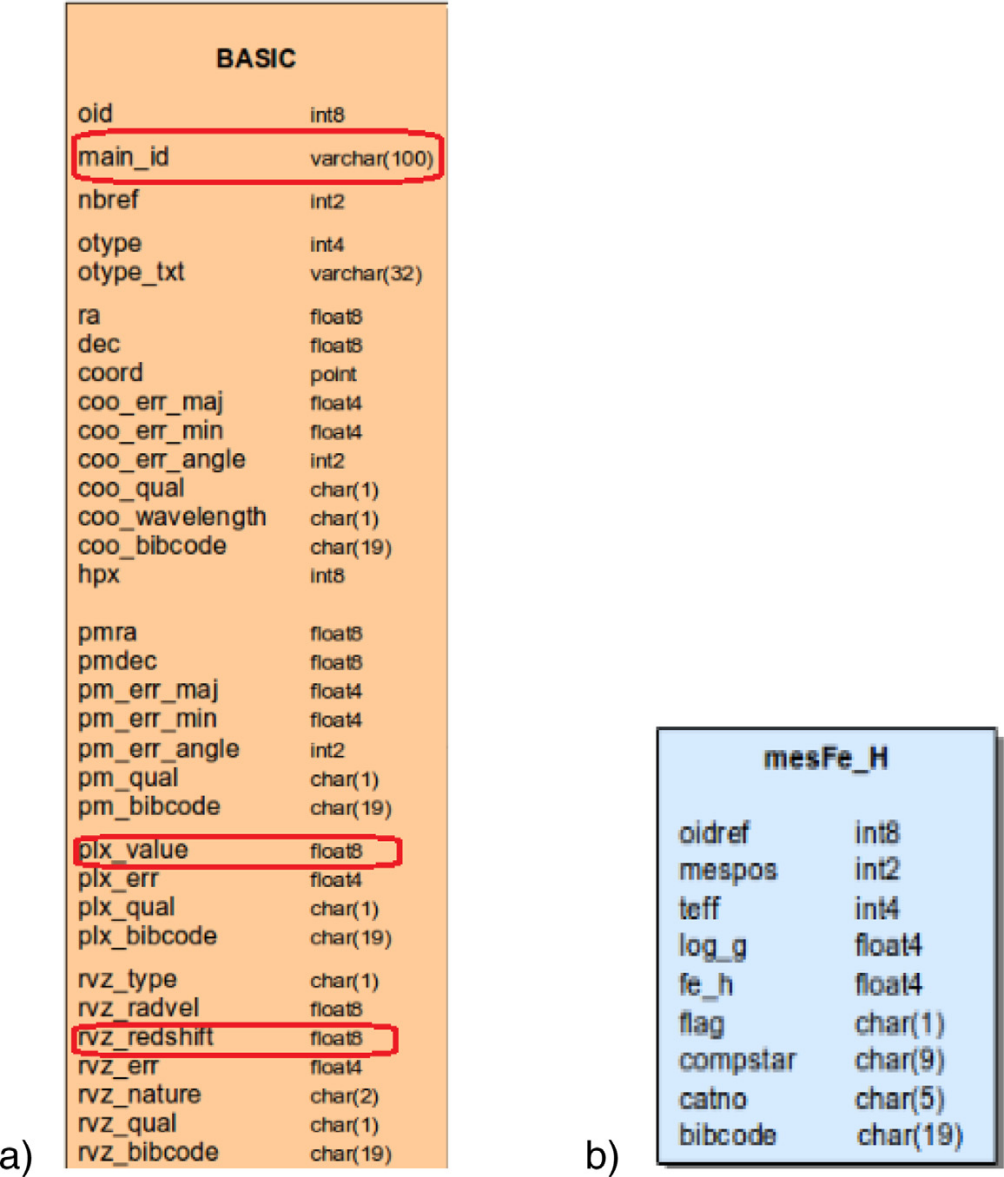
Distribution of 78 high Redshift nearby objects with temperature between 4000 and 14000 K.

Programmers need to know the name and structure of the tables and the name of the fields. The TAP Service provides this information on a separate page. At the bottom-left of the Fig. 3 click the “Simbad table” button or use its direct link to see tables and their fields: https://simbad.cds.unistra.fr/simbad/tap/tapsearch.html.

The Simbad database is a relational database. At the top of each table, its name is written. Fig. 4.a shows parameters object name (*main_id*), distance (*plx_value*), and Redshift (*rvz_redshift*) are in the BASIC table. Also, Fig. 4.b shows that parameter temperature (*teff*) is in the *mesFe_h* table.

The main body of the code is lines 2-5. These lines are written in the standard query language. In Line 2 we can introduce the number of records and parameters that we need to retrieve their data. In Line 3 and 4, the relationship and sequence of tables are introduced. Finally, in Line 5, conditions for filtering output data have been introduced. By applying some conditions to the script, the result can be filtered.

We have used three conditions:

1. *plx_value* >1 limits the output records to close space objects for using more precise distances.
2. *rvz_redshift* >0 prevents space objects with the Blueshift.
3. *teff* >100 prevents having space objects with unknown temperatures or less than 100 K.

We can copy and paste them into the Tap service and run it by clicking the start button. Here are the codes altogether:

SELECT top 20000 main_id, plx_value, rvz_redshift, teff

FROM basic JOIN ident ON oid=ident.oidref

LEFT JOIN mesFe_h ON oid=mesFe_h.oidref

WHERE plx_value >1 and rvz_redshift >0 and  teff>100

For retrieving more data, we can use different temperature limits and increase them in each execution such as (*teff*>100 and *teff*<1000) then (*teff*>=1000 and *teff*<1300), and so on. On the other hand, we can change *rvz_redshift* >0 to *rvz_redshift* <0 for retrieving blueshift data or delete it for having all space objects together.

## Ethics Statements

This study does not involve human participants and samples derived from human. It also does not involve animals including live vertebrates and higher invertebrates.

## CRediT authorship contribution statement

**Bahram Kalhor:** Methodology, Software, Investigation, Validation, Writing – review & editing.

## Declaration of Competing Interest

The authors declare that they have no known competing financial interests or personal relationships that could have appeared to influence the work reported in this paper.

## Data Availability

Nearby Space Objects Name_Distance_ Redshift_Temperature (Reference data) (figshare.com).
